# Supplementary material for: Sedentary patterns and health outcomes in the oldest-old: a latent profile analysis
Source: PeerJ. 2024 Jun 24;12:e17505. doi: 10.7717/peerj.17505 (PMC11210487; doi:10.7717/peerj.17505)
Supplement: Supplemental Information 3 [file peerj-12-17505-s003.pdf]

Codebook Healthy 80+ study

Categorical Data	Code	Respective Factor	-> Recoded Categorical Data	Code	Respective Factor
Sex	1	Men			
	2	Female			
Familysituation	1	Single			
	2	Partner, living alone			
	3	Cohabiting/married			
House	1	Home			
	2	Service flat			
	3	Nursery home			
Home environment	1	Countryside			
	2	Village			
	3	Outskirts			
	4	City			
Diploma	1	No diploma	-> Education	1	Without higher education (1 -> 5)
	2	Primary school		2	Higher education (6 -> 7)
	3	Vocational secondary education			
	4	Technical secondary education			
	5	General secondary education			
	6	Higher education, no university			
	7	University			
BMI groups	1	Underweight			
	2	Normal range			
	3	Overweight			
	4	Obese			
